# Clove Essential Oil Pickering Emulsions Stabilized with Lactoferrin/Fucoidan Complexes: Stability and Rheological Properties

**DOI:** 10.3390/polym15081820

**Published:** 2023-04-07

**Authors:** Xiaohong Xi, Zihao Wei, Yanan Xu, Changhu Xue

**Affiliations:** 1 College of Food Science and Engineering, Ocean University of China, Qingdao 266404, Chinaxuech@ouc.edu.cn (C.X.); 2 Laboratory of Marine Drugs and Biological Products, Pilot National Laboratory for Marine Science and Technology, Qingdao 266237, China

**Keywords:** lactoferrin, fucoidan, Pickering emulsion, clove essential oil, stability, rheological property

## Abstract

Although studies have shown that lactoferrin (LF) and fucoidan (FD) can be used to stabilize Pickering emulsions, there have been no studies on the stabilization of Pickering emulsions via the use of LF–FD complexes. In this study, different LF–FD complexes were obtained by adjusting the pH and heating the LF and FD mixture while using different mass ratios, and the properties of the LF–FD complexes were investigated. The results showed that the optimal conditions for preparing the LF–FD complexes were a mass ratio of 1:1 (LF to FD) and a pH of 3.2. Under these conditions, the LF–FD complexes not only had a uniform particle size of 133.27 ± 1.45 nm but also had good thermal stability (the thermal denaturation temperature was 110.3 °C) and wettability (the air-water contact angle was 63.9 ± 1.90°). The concentration of the LF–FD complexes and the ratio of the oil phase influenced the stability and rheological properties of the Pickering emulsion such that both can be adjusted to prepare a Pickering emulsion with good performance. This indicates that LF–FD complexes represent promising applications for Pickering emulsions with adjustable properties.

## 1. Introduction

The increase in the interfacial area between the two phases in an emulsion leads to the rapid aggregation of droplets and phase separation. Therefore, emulsions are considered to be thermodynamically unstable systems [1]. Usually, the interfacial tension of conventional emulsions can be reduced by using surfactants. However, the adsorption of surfactants at the oil–water interface is reversible, which has led to the discovery of Pickering emulsions, which have attracted widespread interest [2]. Pickering emulsions refer to emulsions stabilized by solid colloidal particles instead of conventional emulsifiers [3]. Due to the special interfacial solid layer of a Pickering emulsion and its spatial site resistance effect, the particles are irreversibly adsorbed at the interface, leading to better stability [2,4]. In addition, the emulsifier used to stabilize Pickering emulsions is small and widely available. For example, maize starches were modified through a media milling process to form nano/sub-micrometer-milled starch particles [5]. It was shown that the stability of a Pickering emulsion stabilized with these milled starch particles increased with the increase in grinding time. Thus, Pickering emulsions are considered a safer, less toxic, and lower-cost particle-stabilized emulsion system.

For safety and environmental reasons, many scholars in the field of food have chosen natural biomolecules, such as proteins and polysaccharides, to obtain solid particles for stabilizing Pickering emulsions. For example, the preparation of an O/W Pickering emulsion stabilized with chitosan particles via one-step emulsification with an ultrasonic device [6]. Emulsions that are stabilized with soft whey protein microgel particles have good biocompatibility and a high degree of resistance to coalescence [7]. Protein-based particles are of great interest to researchers due to their excellent properties, such as amphiphilicity, nontoxicity, biocompatibility, biodegradability, and natural abundance [8,9]. However, protein particles’ high aggregation tendency and relatively poor structural integrity make their application somewhat limited [10]. Therefore, polysaccharides are usually used to modify proteins in order to change their structure and function so that the resulting particles can better stabilize Pickering emulsions [11]. Similarly, the poor surface activity and emulsification properties of polysaccharides could be improved by protein modifications [10]. It has been shown that Pickering emulsions stabilized with pea protein isolate (PPI) and high methoxylated pectin (HMP) composite particles have higher stability to pH changes and higher retention of β-carotene compared to PPI-stabilized high internal phase Pickering emulsions [12]. For this reason, there are increasing studies on protein–polysaccharide complexes for stabilizing Pickering emulsions.

Lactoferrin (LF) is a naturally occurring iron-binding protein with a high isoelectric point (pH 8.0–9.0). As a multifunctional protein, LF has a variety of biological activities, including antibacterial, antioxidant, anti-inflammatory, and anticancer [13,14,15,16]. However, single LF-stabilized emulsions are prone to destabilization under extreme environmental conditions, such as high heat, high salt, strong acid, and strong alkali, near the isoelectric point, which can be overcome by forming complexes with polysaccharides. Fucoidan (FD), mainly containing L-fucopyranose units and sulfated ester groups, is a water-soluble polysaccharide with various biological activities, such as antioxidant, anti-inflammatory, and anticancer [17,18,19,20]. Due to the negatively charged sulfate groups, good solubility, and a high charge density over a wide pH range, FD can form stable complexes with other positively charged molecules [19]. Therefore, when the reaction system is below the isoelectric point of LF, the positively charged LF and the negatively charged FD can form an electrostatic complex due to electrostatic interaction. It has been shown that the thermal denaturation of proteins can be inhibited by binding to polysaccharides. For instance, complexes obtained by mixing LF and lactose in a specific ratio and heating has better iron retention and binding capacity than heated LF [21].

To the best of our knowledge, there are no reports on Pickering emulsions that have been stabilized with LF–FD complexes. In this study, we used negatively charged FD and positively charged LF to form LF–FD complexes via electrostatic interactions to prepare Pickering emulsions with clove essential oil as the oil phase. The structural characteristics of the LF–FD complexes were characterized by zeta potential, mean particle size, Fourier transform infrared spectroscopy (FTIR), and scanning electron microscopy (SEM). Subsequently, the stability and rheological properties of the Pickering emulsions were evaluated.

## 2. Materials and Methods

### 2.1. Materials

Lactoferrin (LF) was purchased from Hilmar Ingredients (Hilmar, CA, USA). Fucoidan (FD) was provided by Qingdao Bright Moon Seaweed Group Co., Ltd. (Qingdao, China). Clove essential oil was obtained from Sinopharm Chemical Reagent Co., Ltd. (Shanghai, China). Unless otherwise specified, all other chemicals or reagents were of analytical grade and obtained from Sinopharm Chemical Reagent Co., Ltd. (Shanghai, China).

### 2.2. Preparation of LF–FD Complexes

LF and FD were dissolved in distilled water at room temperature to obtain solutions of 0.2 wt% concentration and then hydrated overnight at 4 °C to ensure the adequate dissolution of LF and FD. The FD solution was diluted in different gradients so that the diluted solution was mixed with the LF solution in equal volumes to obtain the mixed solution. The final mass ratios of LF to FD were 1:1, 1.5:1, and 2:1, respectively. The pH of the mixed solution was adjusted to the envisaged values (1.5, 2, 2.5, 3, 3.5, 4, and 4.5) with 1 M NaOH and 1 M HCl solution, and the LF–FD solution was stirred magnetically at room temperature for 2.5 h. LF–FD solution was heated and stirred for 15 min in a water bath at 95 °C. After the heat treatment, the solution was rapidly cooled to room temperature to prevent further denaturation.

### 2.3. Characterization of LF–FD Complexes

#### 2.3.1. Determination of Turbidity

The transmittance (T) of LF–FD complexes solution at λ = 600 nm under different conditions was determined by a spectrophotometer (UV 2355, Unico, Shanghai, China) at 25 °C. Each sample was measured in parallel three times, and the average value was taken. The turbidity values were corrected by using de-ultrapure water as a blank control. The turbidity was expressed as 100-T% [22].

#### 2.3.2. Particle Size, Polydispersity Index (PdI) and Zeta Potential

The particle size, PdI, and zeta potential of the LF–FD complexes were measured by a Zeta sizer Nano ZS90 (Malvern^®^ Instruments, Malvern, UK) to avoid the negative influence of multiple scattered light effects caused by high concentration samples on the measurement results. The sample should be diluted to 1 mg/mL before measurement. The measurement temperature was 25 °C, and the equilibration time was 120 s. Each sample was measured three times in parallel, and the average value was taken.

#### 2.3.3. Analysis of LF–FD Complexes Micromorphology

The morphology of LF–FD complexes was analyzed by the scanning electron microscope (S-4800 Hitachi, JEOL, Tokyo, Japan) at an accelerating voltage of 20 kV. The lyophilized samples are fixed on double-sided tape for gold coating.

#### 2.3.4. Determination of Contact Angles

The air-water contact angle (*θ*_aw_) of LF–FD complexes was measured by an optical contact angle meter measuring instrument (OCA 15EC, Dataphysics, Filderstadt, Germany) as a basis for judging the wettability of complexes. Specifically, the prepared samples were lyophilized to obtain sample powder, which was pressed into thin tablets of about 1–2 mm by a tablet press. The *θ*_aw_ was measured by taking an image of the droplet using a camera after injecting 3 μL of ultrapure water on the surface of the tablet with a syringe.

#### 2.3.5. Analysis of Fourier Transform Infrared (FTIR) Spectroscopy

The FTIR spectra of LF–FD complexes were characterized by FTIR (Nicolet iS10, Thermo, Waltham, MA, USA) from 4000–400 cm^−1^ with a resolution of 4 cm^−1^ and 32 scans. Specifically, an appropriate amount of lyophilized sample was ground to a fine powder in an agate mortar and then mixed with dried potassium bromide (KBr) powder. And the tablet of moderate thickness was obtained by a tablet press.

#### 2.3.6. Analysis of Differential Scanning Calorimetry (DSC)

Under a nitrogen atmosphere, the samples were analyzed for thermal stability using differential scanning calorimetry (DSC3 Differential Scanning Calorimeter, Mettler Toledo, Greifensee, Switzerland). The samples were placed in hermetic pans and heated from 30 °C to 200 °C at a constant temperature rate of 10 °C/min.

### 2.4. Preparation of Pickering Emulsions

The LF–FD complex solutions with a mass ratio of 1:1 were prepared according to the above method. Briefly, LF and FD solutions of different concentrations (0.5 wt%, 1 wt%, 1.5 wt%, and 2 wt%) were mixed in equal volumes after overnight hydration at 4 °C, and the pH of the mixed solutions was adjusted to 3.2 with 1 M NaOH and HCl solutions and then magnetically stirred for 3.5 h. The LF–FD solutions were magnetically stirred in a water bath at 95 °C for 15 min and rapidly cooled to room temperature at the end of the heat treatment. Finally, different concentrations of LF–FD complex solution and clove oil were mixed in different oil phase ratios (0.3, 0.5, 0.6, and 0.65). Then, the mixture was sheared for 3 min at 13,200 rpm/min by a T25 digital Ultra Turrax (IKA, Stauffen, Germany) to obtain Pickering emulsions.

### 2.5. Characterization of the Pickering Emulsions

#### 2.5.1. Type of Emulsion, Appearance, and Droplet Size

In this paper, we used the dilution method to determine the type of emulsion. Specifically, the emulsion was dropped into water or oil, and its dispersion in water and oil was observed. If the emulsion droplets could be evenly dispersed in water, the emulsion was an oil-in-water emulsion; conversely, if the emulsion droplets could not be dispersed in water but could be dispersed in oil, the emulsion was an oil-in-water emulsion [23].

The appearance of the prepared Pickering emulsion was photographed with a camera. The droplet size and size distribution of Pickering emulsions were determined using a laser particle sizer (Bettersize 2600, Dandong Bettersize Instrument Co., Ltd., Dandong, China).

#### 2.5.2. Visual Assessment of Morphology

The droplet morphology, size, and degree of aggregation of the prepared Pickering emulsions were observed under a bright field. To further verify the adsorption of LF–FD complexes at the oil–water interface, the protein and oil phase were first stained with Rhodamine B and Nile Red, respectively. Then the Pickering emulsion was observed using an electric fluorescence microscope (ECLIPSE Ni-E, Nikon Corporation, Tokyo, Japan).

### 2.6. The Stability of Pickering Emulsions

#### 2.6.1. Storage Stability

Immediately after emulsion preparation, the same volume of the emulsion was transferred to a clear glass sample bottle, and the appearance of the emulsion was compared at 2 h, 7 d, 15 d, and 30 d to determine the stability of the emulsions prepared under different conditions by the Emulsion Stability Index (*ESI*). The *ESI* was calculated by the following equation:

(1) $$ESI\left(\%\right)=\frac{H_{E}}{H_{T}}\times 100\%$$

where *H*_E_ is the height of the emulsion phase, and *H*_T_ is the total height of emulsions.

#### 2.6.2. Centrifugal Stability

After the preparation of the emulsion, the same volume of the emulsion was taken into a centrifuge tube, and the emulsion was centrifuged at 5000 rpm for 5 min. After centrifugation, the volume of the different phases separated from the emulsion was observed to compare the centrifugal stability of the prepared emulsion under different conditions.

#### 2.6.3. Turbiscan Stability Index (TSI) and Difference in Backscattered Light (ΔBS)

The stability of LF–FD stabilized Pickering emulsions were evaluated by the TurbiscanLAB instrument (Formulaction, Smart Scientific Analysis, Toulouse, France). The emulsions of 10 mL were transferred to a special test bottle and then placed in the instrument for scanning [24]. The scanning temperature was 25 °C, and the scanning time was 2 h.

### 2.7. Rheological Properties of Pickering Emulsions

The rheological properties of Pickering emulsions were measured by the MCR 301 rheometer (AntonPaar GmbH, Graz, Austria). Measurements were performed using a 50 mm conical parallel plate (CP 50) with a gap of 1 mm at a measurement temperature of 25 °C. The apparent viscosity of the emulsions was determined over a shear rate range of 0–100 s^−1^. Stress scans were performed to determine the deterministic viscoelastic region in the range of 0.1–100 Pa at a fixed frequency of 1 Hz. The strain was fixed at 0.1%, and the frequency scans were performed in the range of 1–20 Hz to obtain the storage modulus (G′) and shear modulus (G″) with frequency. A power law model could be used to fit a nonlinear regression to the apparent viscosity (*γ*) of the emulsion.

(2) $$\tau =K\gamma^{n-1}$$

where *τ* is the shear stress, *K* is the viscosity coefficient, and *n* is the fluid index.

### 2.8. Statistical Analysis

Each experiment was conducted in triplicate at least. SPSS 25.0 (SPSS Inc., Chicago, IL, USA) was used to determine the statistical differences (*p* < 0.05) through analysis of variance (ANOVA) and Duncan’s test. All Graphs were created with Origin 2023 (OriginLab Co., Northampton, MA, USA).

## 3. Results and Discussion

### 3.1. Characterization of the LF–FD Complexes

#### 3.1.1. Effect of pH on LF–FD Interactions

**Figure 1:**
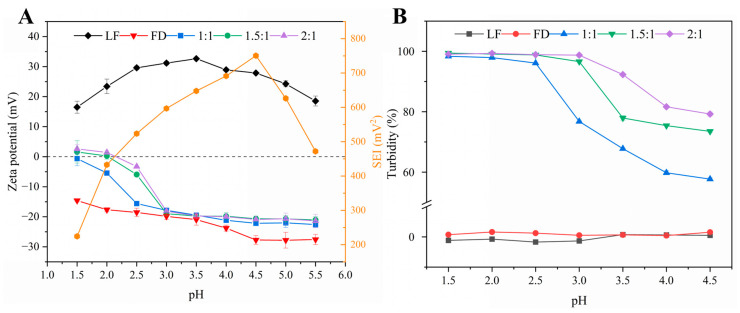
The zeta potential, SEI (**A**), and turbidity (**B**) of LF, FD, and the LF–FD complexes (1:1, 1.5:1 and 2:1) as a function of pH.

Protein-polysaccharide complexes are usually formed by electrostatic interactions, and adjusting the pH to change the charge density on the surface of the macromolecule is a common method to influence the strength of electrostatic interactions. As an anionic polyelectrolyte, FD was always negatively charged throughout the pH range (1.5–5.5) (as shown in Figure 1A). The zeta potential of FD decreased with increasing pH at pH 1.5–4.5, which was related to the pKa value of the carboxylic acid molecule [25]. The zeta potential of FD remained basically unchanged at pH 4.5–5.5, which was about −27.7 mV. LF is a cationic protein with an isoelectric point between 8 and 9 [26], so LF was always positively charged throughout the pH range (1.5–5.5). From Figure 1A, the zeta potentials of LF–FD (1:1, 1.5:1, and 2:1) all decreased with increasing pH and then remained basically unchanged, which was roughly similar to the trend of FD, indicating that the surface of the LF–FD complexes was rich in FD chains and the core of the complexes was composed of LF molecules [21]. In addition, the isoelectric points of the LF–FD complexes obtained at different mass ratios ranged from 1.5 to 2, which were significantly lower compared to the isoelectric points of LF (pH 8–9). The apparent change in the isoelectric point might be due to the attachment of the polysaccharide to the exterior of the protein and the decrease in the isoelectric point of the complexes caused by the electrostatic interaction between LF and FD.

The strength of electrostatic interaction (SEI) can show the difference in zeta potential between the polyelectrolytes. In the pH range where the zeta potential difference is large, there is a strong electrostatic interaction between the polyelectrolytes [27]. SEI is expressed using the absolute value of the product of the zeta potentials of those polyelectrolytes with opposite charges at different pH values [25]. As shown in Figure 1A, the strongest electrostatic interaction between LF and FD occurred in the pH range of 4.5–5.0, and SEI had a maximum value at pH 4.5, indicating that the strongest electrostatic gravitational force was generated between LF and FD under this condition.

From Figure S1, there was a significant change in the turbidity of the LF–FD complexes before and after heating. Specifically, the unheated LF–FD solution showed high transparency and almost no visual change under different pH conditions, while the heated LF–FD solution became significantly turbid, and the turbidity tended to be obvious as the pH decreased. Turbidity reflects the size and number of particles and can be used to analyze the formation of complexes [22].

The effect of different pH on the turbidity of LF, FD, and the LF–FD complexes (1:1, 1.5:1, and 2:1) can be seen in Figure 1B. The turbidity of LF and FD remained almost constant with pH, while the turbidity of the heated LF–FD complex solution decreased with increasing pH and then remained basically unchanged. Among them, the turbidity of the LF–FD complex (1:1) tended to be 100% in the pH range of 1.5–2.5, while the LF–FD complex (1.5:1 and 2:1) had a trend of 100% in the pH range of 1.5–3.0. The turbidity variation in this pH range was caused by electrostatic interactions due to the charge characteristics of LF and FD. When compared with the LF–FD complex (1.5:1 and 2:1), the maximum turbidity value of the LF–FD complex (1:1) was left-skewed, which was caused by the fact that the FD was negatively charged throughout the pH range, and the rise in its content increased the net negative charge of the solution. According to Figure S2, pH_opt_ and pH_φ_ were the critical pH transition points. The displayed pH range was mainly in the region of insoluble complexes, and the turbidity decreased abruptly during the increase in pH to 4.0, indicating that the insoluble complexes started to dissociate to form soluble complexes when the critical point pH_φ_ was reached [28]. When pH < pHφ, there was precipitation in the solution (as shown in Figure S1), indicating that phase separation started to occur in the system. In addition, the turbidity increased sharply with decreasing pH, and the maximum value appeared at pH_opt_. The strongest electrostatic interaction between the two polyelectrolytes occurred at pH_opt_, which was because the opposite charges carried by the polyelectrolytes at this time canceled each other out, resulting in the complex being electrically neutral [29]. This was also evidenced by the zeta potential of the LF–FD complex.

#### 3.1.2. Effect of pH on Particle Size

**Figure 2:**
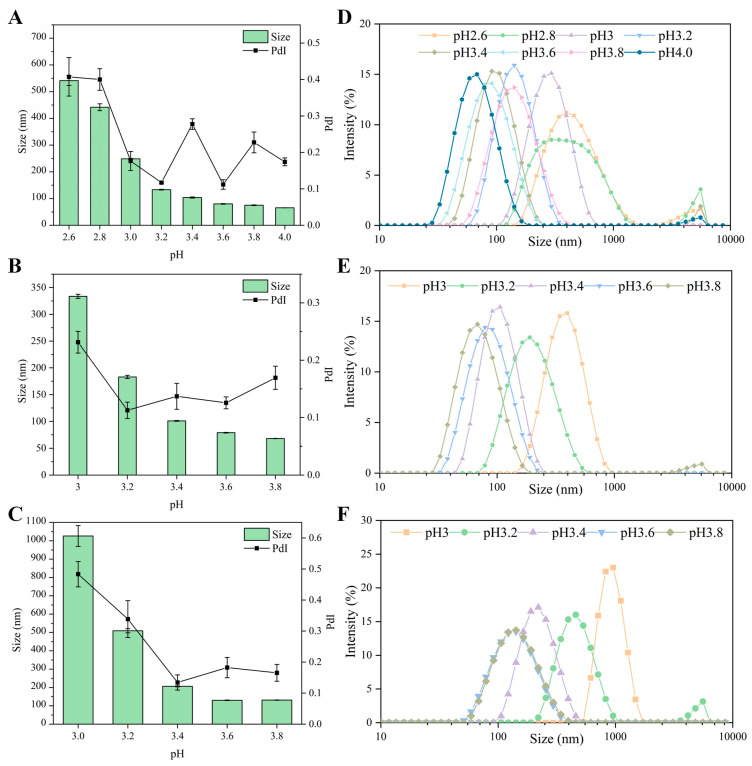
The particle size, polydispersity index (PdI) (**A**–**C**) and particle size distribution (**D**–**F**) of LF–FD complexes with different ratios (1:1, 1.5:1 and 2:1).

Based on the above results, the particle size of the LF–FD complexes was investigated. Figure 2A–C show the average particle size of the LF–FD complexes at different pH values. The mean particle size of the LF–FD complexes decreased with increasing pH. The particle size of the particles is critical for their emulsification ability and the stability of Pickering emulsions. In general, the smaller the particle size, the greater the diffusion rate and accumulation effect of the particles at the oil–water interface [30]. Nevertheless, it has also been shown that the smaller the particle size, the less adsorption energy is required to detach at the interface, making the emulsion easy to break [24]. Meanwhile, the particle size should not be too large, and particles with a particle size exceeding 500 nm may cause precipitation in a solution [31]. Therefore, the particle size should not be too large or too small.

From Figure S1, it can be seen that when the mass ratio of LF to FD was 1:1, there was more obvious precipitation at the bottom of the heated LF–FD complex solution at pH 2.6 and 2.8, while the LF–FD complex solution at mass ratios of 1.5:1 and 2:1 also had a little precipitation at pH 3.0, which was consistent with the corresponding larger particle size. Figure 2D–F show the particle size distribution and the wide particle size distribution implied an uneven particle size, which was detrimental to the stability of the Pickering emulsion. Taking the LF–FD complexes (1:1, pH 3) as an example, although their average particle size was 248.67 ± 1.32 nm, more than 20% of the complex had a particle size above 400 nm, which was unevenly dispersed compared to other particles. Since the smaller the PdI, the more homogeneous the particle dispersion, the LF–FD complexes (1:1, pH 3.2), LF–FD complexes (1.5:1, pH 3.4), and LF–FD complexes (2:1, pH 3.4) were selected for the follow-up experiments, and the corresponding mean particle sizes were 133.27 ± 1.45 nm, 100.93 ± 0.74 nm, and 206.17 ± 2.73 nm, respectively; the PdI was 0.117 ± 0.003, 0.137 ± 0.023 and 0.134 ± 0.025, respectively. For the convenience of writing, the mass ratio of LF and FD was used to refer to the LF-FD complex in subsequent experiments, e.g., LF–FD complex (1:1) instead of LF–FD complex (1:1, pH 3.2).

#### 3.1.3. The Micromorphology Characters of LF–FD Complexes

**Figure 3:**
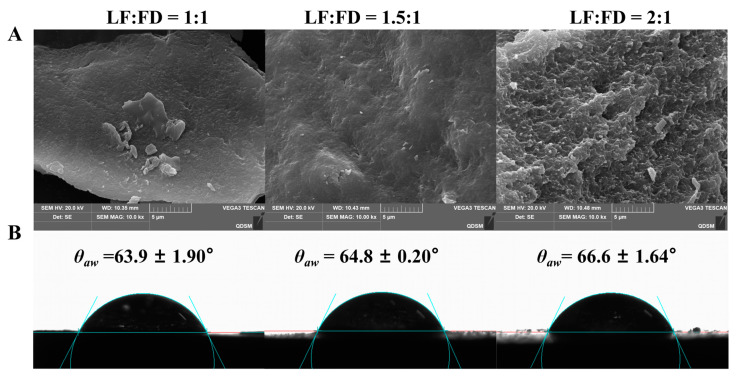
SEM micrographs (**A**) and static water-in-air contact angles (**B**) of the LF–FD complexes.

Figure 3A shows the SEM images of the LF–FD complexes prepared with different mass ratios. As the percentage of FD decreased, the irregular particle structure in the SEM images of the LF–FD complexes gradually became obvious (caused by the LF spherical structure), and the sample surface tended to be rough [32]. Most of the polysaccharides were chain-like structures, which would appear “sponge-like” after water loss caused by freeze-drying treatment [33]. Therefore, the lyophilized complexes also exhibited irregularly arranged cavities structures. Therefore, the decrease in FD concentration caused a sparser arrangement of cavities in the lyophilized samples. Since FD was located on the surface of the LF–FD complexes, the reduction in the FD percentage made the irregular particle structure on the surface of LF–FD complexes more obvious.

#### 3.1.4. Contact Angle Analysis of the LF–FD Complexes

In addition to particle size, the wettability of the particles also plays an important role in the stability of a Pickering emulsion [34]. The wettability of the particles is usually reflected by measuring the contact angle, and Figure 3B shows the air-water contact angle (*θ*_aw_) of the LF–FD complexes prepared with different mass ratios. When *θ*_aw_ was in the range of 53°–82°, the particles had intermediate wettability and could be used to stabilize the Pickering emulsions [35]. Those particles with intermediate wettability could quickly form an interfacial layer at the oil–water interface to prevent oil droplets from aggregating. The *θ*_aw_ of the LF–FD complexes with different mass ratios (1:1, 1.5:1, and 2:1) were 63.9 ± 1.90°, 64.8 ± 0.20°, and 66.6 ± 1.64°, respectively, which were significantly lower compared to a *θ*_aw_ of 129.2 ± 3.29° for the heated LF (Figure S3). This indicated that the three LF–FD complexes could be used to stabilize O/W Pickering emulsions [36]. In addition, the increase in the proportion of FD resulted in a corresponding decrease in the *θ*_aw_ of LF–FD complexes. Obviously, the addition of FD significantly improved the wettability of the heated LF, resulting in the enhanced hydrophilicity of the LF–FD complex. This was because the hydrophilic FD was attached to the LF surface, increasing the hydrophilic groups on the surface of the complex. Similar phenomena were observed in other protein–polysaccharide complexes, such as the modification of hydrophobic zein by hydrophilic pectin to obtain zein–pectin particles, which showed a significant increase in the hydrophilicity of the particles [37]. The above results showed that the LF–FD complexes (1:1, 1.5:1, and 2:1) could all be used to stabilize O/W Pickering emulsions. Furthermore, the *θ*_aw_ of the LF–FD complex (1:1) was closer to the ideal *θ*_aw_ (59°), indicating that it had the best ability to stabilize the Pickering emulsion [35].

#### 3.1.5. The FTIR Spectra of the LF–FD Complexes

**Figure 4:**
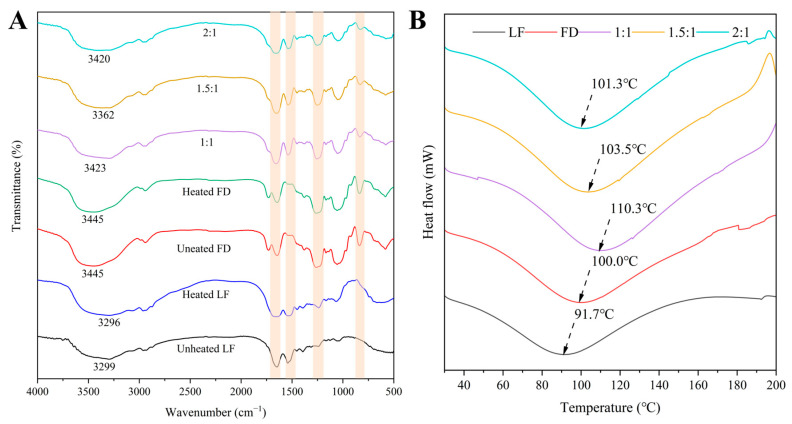
(**A**) FTIR spectra of unheated LF, unheated FD, and the LF–FD complexes (1:1, 1.5:1, and 2:1); (**B**) DSC thermograms of unheated LF, heated LF, unheated FD, heated FD, and the LF–FD complexes (1:1, 1.5:1 and 2:1).

As an effective means to analyze the changes in functional groups of biomolecules, FTIR can be used to analyze the interactions between LF and FD. As shown in Figure 4A, the unheated LF had typical absorption peaks at 3299 cm^−1^ (amide A band), 1651 cm^−1^ (amide I band), 1541 cm^−1^ (amide II band), and 1394 cm^−1^ (amide III band). The amide A band mainly consists of hydrogen bonding and N–H stretching vibrations, and the amide I band mainly represents the stretching vibration of C=O. The amide II band represents the C–N stretching vibration and N–H planar bending vibration, and the amide III band is the stretching vibration of C–N and N–H groups [38]. The amide A band, amide I band, and amide II band of LF were slightly red-shifted after heating, which indicated that heating caused changes in the amide bond conformation and intramolecular hydrogen bonding of LF. The unheated FD had significant absorption peaks at 3445 cm^−1^ (O–H stretching vibration), 1261 cm^−1^, and 837 cm^−1^, with the latter two being the characteristic peaks of the FD sulfate group, which are associated with O=S=O stretching and C–O–S bending vibration [19,39]. There was no change in the peaks of the heated FD, indicating that heating did not significantly affect the structure of the FD. The peaks associated with the amide A, amide II bands, and amide III bands were found to be significantly blue-shifted for the LF–FD complexes (1:1, 1.5:1, and 2:1) compared to the heated LF, while the characteristic peaks of the sulfate group of FD were found to be slightly red-shifted and increased in intensity with the increase in the percentage of FD, indicating the bonding of –NH_2_ on LF with the sulfate group on FD through electrostatic interactions [32].

#### 3.1.6. The DSC Analysis of the LF–FD Complexes

Generally, DSC was used to evaluate the thermal stability of the samples. As shown in Figure 4B, the thermal denaturation temperatures of the unheated LF and unheated FD were 91.7 °C and 100.0 °C, respectively, which were consistent with the results of related studies [32,39]. The thermal denaturation temperatures of the LF–FD complexes increased with the increase in the percentage of FD and were higher than those of the unheated LF and unheated FD. This could be because the addition of FD improved the hydrophilicity of the heated LF, thus enhancing the thermal stability of the LF–FD complexes. The results of the contact angle proved that the addition of FD enhanced the hydrophilicity of the LF-FD complexes and was positively correlated with the FD content. The LF–FD complex (1:1) had the largest thermal denaturation temperature of 110.3 °C compared to the other ratios of the LF–FD complexes, which was 18.6 °C higher than that of the unheated LF. According to the FTIR results, this could be caused by the presence of electrostatic and other noncovalent interactions during the formation of the LF–FD complexes, resulting in the enhanced thermal stability of the complex. Studies showed that the enhancement of protein thermal stabilization could make the tertiary structure of proteins more stable, which was conducive to improving the emulsification properties of proteins [40]. In conclusion, the LF–FD complex (1:1) had the proper size and good wettability, thermal stability, and emulsification properties. Therefore, the LF–FD complex (1:1) was selected for the preparation of the Pickering emulsions required for the subsequent experiments.

### 3.2. Characterization of Pickering Emulsions

#### 3.2.1. Type of Emulsion

Figure S4 shows the dispersion of a Pickering emulsion stabilized by the LF–FD complex (1:1) in the water and oil phases. The emulsion was found to be largely dispersed in the water and suspended in the oil phase, which is a typical O/W-type emulsion, and this was consistent with the above contact angle measurement results.

#### 3.2.2. Storage Stability

The storage stability was determined by examining whether the emulsion appeared to delaminate or disintegrate over time during long-term storage. The changes in the appearance of the Pickering emulsions with different oil-to-water ratios stabilized by different concentrations of the LF–FD complexes after storage for 2 h, 7 d, 15 d, and 30 d can be observed in Figure S5. The emulsion creamed easily at the beginning of storage, making it difficult to accurately evaluate the stability of emulsions. Therefore, the emulsions were selected to be measured after 2 h of resting. As the density of clove essential oil is 1.039–1.051 g/mL, which is slightly greater than the density of water (1 g/mL), the upper layer of the emulsion represents the precipitated water phase. Under the same concentration conditions, the volume of the emulsion phase increased in accordance with the increase in the oil phase ratio (*φ* = 0.3–0.65). Under the same oil phase conditions, the volume of the emulsion phase also increased with the increase in the LF–FD complex concentration (*c* = 0.5–2 wt%). It could be concluded that when the complex concentration was fixed, a higher oil phase ratio could lead to an increase in the volume of the emulsion phase of the Pickering emulsion.

The changes in the *ESI* of the emulsions were consistent with those observed visually (Table 1). Moreover, the *ESI* of the emulsions (*φ* = 0.6–0.65) was above 90% at *c* = 1.5–2 wt%, indicating that the Pickering emulsions stabilized with the LF–FD complexes were stable during storage. Usually, storage stability experiments for Pickering emulsions require the addition of sodium azide to prevent the growth and multiplication of micro-organisms, for example, Pickering emulsions stabilized with whey isolate protein-short-chain inulin and glycosylated whey protein isolate/cyanidin-3-glucoside and Pickering emulsions stabilized with zein–gallic acid composite nanoparticles [41,42]. Both were supplemented with sodium azide to inhibit microbial growth and reproduction in the storage experiments. The Pickering emulsion that was stabilized with the LF–FD complexes without the addition of sodium azide had good stability that remained for 30 days of storage. This was due to the antimicrobial properties of the Pickering-emulsion-embedded clove essential oil, which inhibited the growth of micro-organisms. In addition, the LF–FD complexes were prepared at pH 3.2, and microbial growth was inhibited in this pH environment [22].

**Figure 5:**
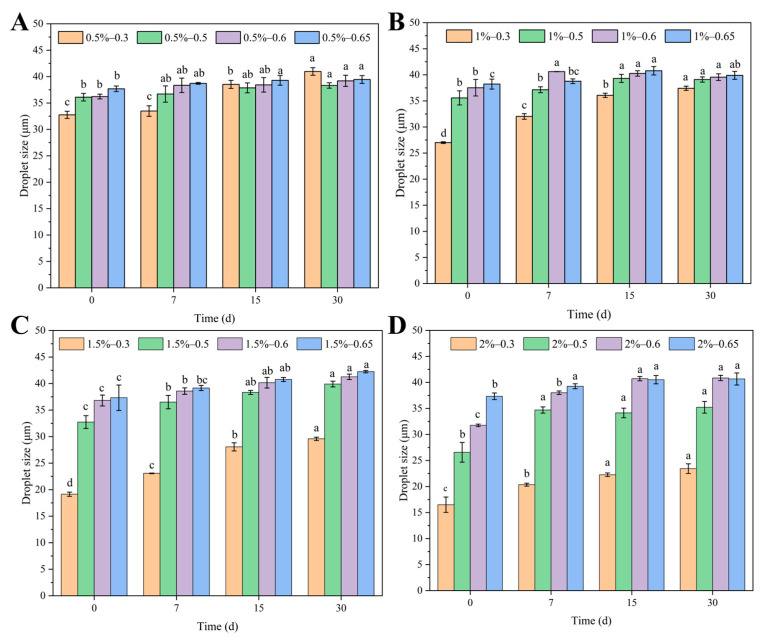
Droplet size of Pickering emulsions stabilized with different *c* (0.5–2 wt%) of LF–FD complexes at different *φ* (0.3–0.65): (**A**) *c* = 0.5 wt%; (**B**) *c* = 1 wt%; (**C**) *c* = 1.5 wt%; (**D**) *c* = 2 wt%. The different letters represent significant differences (*p* < 0.05).

In addition to *ESI*, changes in the droplet size of an emulsion can also reflect its stability during storage. Smaller emulsion droplet sizes result in larger specific surface areas and more orderly particle reorganization, making the emulsions more stable [43]. As shown in Figure 5A,B, the droplet size of the emulsions ranged from 19.14 to 42.24 μm and was negatively correlated with the concentration of the LF–FD complexes. Due to the increased concentration of the complex, more dense interfacial layers could be formed at the oil–water interface to stabilize more emulsion droplets, and the increase in the number of droplets led to a decrease in the emulsion particle size. When the concentration of the LF–FD complex was fixed, the droplet size of the emulsion increased with the oil phase content. In addition, the increase in storage time resulted in a tendency for the oil phase in the emulsion to aggregate, which increased the droplet size.

#### 3.2.3. Centrifugal Stability and Heating Stability

Centrifugal stability was investigated by examining whether emulsion breakage or stratification occurred when the emulsion was subjected to accelerated centrifugal forces. As shown in Figure S6, the emulsified phase increased with the concentration of the LF–FD complex and the oil phase content after centrifugation. Besides, the top of the emulsion showed an obvious water phase, and there was no visible oil phase at the bottom. It indicated that the Pickering emulsion prepared by the LF–FD complex had good centrifugal stability and could encapsulate a high content of clove essential oil [44].

#### 3.2.4. Microstructure Observation

**Figure 6:**
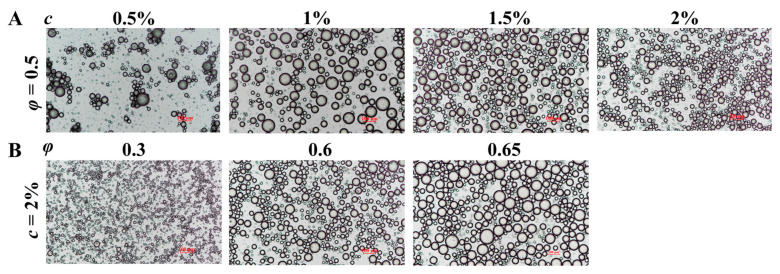
Optical microscopic images of Pickering emulsions stabilized with different *c* (0.5–2 wt%) of the LF–FD complexes at different *φ* (0.3–0.65): (**A**) *φ* = 0.5; (**B**) *c* = 2 wt%. The scale bars within the figures are 100 μm in length.

Figure 6A shows the optical microstructure of Pickering emulsions stabilized with different concentrations (*c* = 0.5–2 wt%) of LF–FD complexes at a fixed oil phase ratio (*φ* = 0.5). The droplet size of the emulsion under the microscope became smaller as the concentration of the complexes increased. At *c* = 0.5 wt%, the emulsion droplets appeared aggregated and extremely heterogeneous. The reason was that the amount of the LF–FD complex was not sufficient to cover the surface of all the oil droplets, resulting in the aggregation of some oil droplets. Figure 6B shows the microstructure of Pickering emulsions with different oil phase ratios (*φ* = 0.3–0.65) stabilized with an LF–FD complex at a fixed complex concentration (*c* = 2 wt%), and the droplet size variation was consistent with that in Figure 5. A portion of the oil droplets in the 2%–0.65 group (*c* = 2 wt%, *φ* = 0.65) of the emulsions formed polygons due to the tight buildup, thus inhibiting some droplet motion. This might be due to the combined effect of LF–FD complex concentration and oil phase ratio increasing the viscosity and droplet size of the emulsion [45].

**Figure 7:**
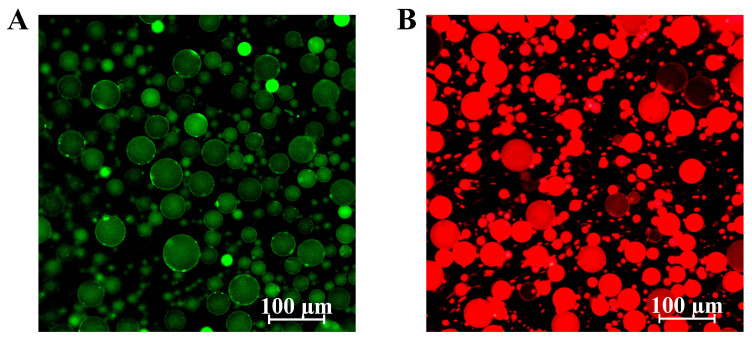
Fluorescence microscopic images of Pickering emulsions stabilized with LF–FD complexes: (**A**) the oil phase stained with Nile Red; (**B**) LF–FD complexes stained with Rhodamine B. The scale bars within the figures are 100 μm in length.

The adsorption of the complex at the water–oil interface was confirmed by fluorescence pictures of the Pickering emulsions. The green fluorescent layer in Figure 7A represents the interfacial layer formed by the LF–FD complexes, and the red circle in Figure 7B represents the oil phase droplets in the emulsion. This demonstrated that the LF–FD complex adsorbed around the oil droplets to stabilize the Pickering emulsion.

#### 3.2.5. TSI

**Figure 8:**
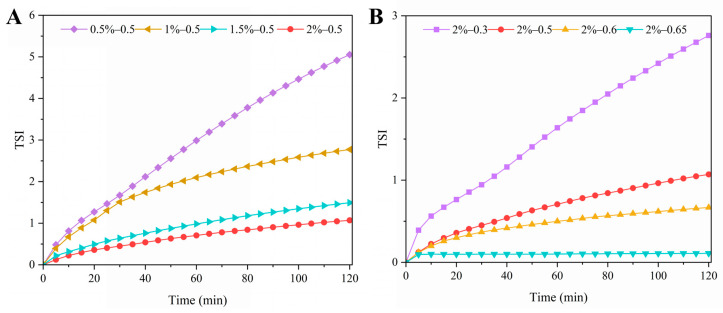
TSI of Pickering emulsions stabilized with different *c* (0.5–2 wt%) of the LF–FD complexes at different *φ* (0.3–0.65): (**A**) *φ* = 0.5; (**B**) *c* = 2 wt%.

TSI was used to evaluate the stability of the Pickering emulsions stabilized with LF–FD complexes. In general, the higher the TSI, the more pronounced the changes in the droplets in the system (flocculation, agglomeration, etc.) and the less stable the emulsion [45]. Figure 8 shows the overall TSI of the Pickering emulsions with different oil–water ratios stabilized by different concentrations of the LF–FD complexes. Except for the TSI of the 2%–0.65 group, which remained basically unchanged, the TSI of all emulsions increased to different degrees with scanning time. Among them, an increasing LF–FD complex concentration led to a decreasing TSI value, with the final TSI value decreasing from 2.8 to 0.3. The higher the ratio of the oil phase at the same concentration, the lower the TSI, with the final TSI value decreasing from 5.1 to 1.1. A lower TSI value indicated less droplet variation and a more stable emulsion system. The above results were the same as the experimental findings for storage stability and centrifugal stability.

#### 3.2.6. ΔBS

**Figure 9:**
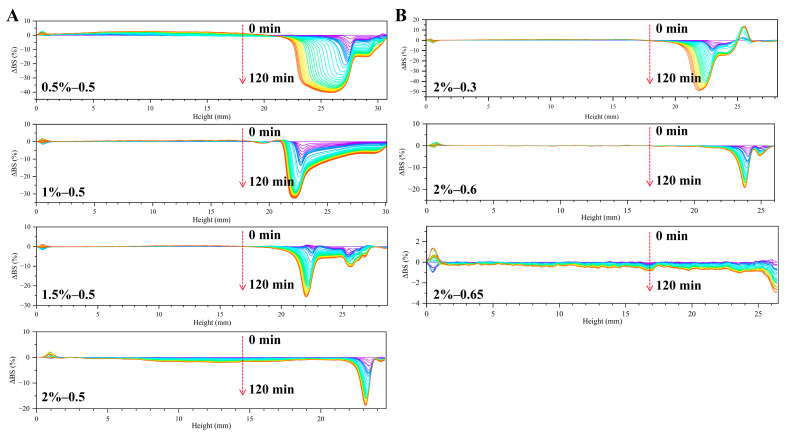
ΔBS of Pickering emulsions stabilized with different *c* (0.5–2 wt%) of LF–FD complexes at different *φ* (0.3–0.65): (**A**) *φ* = 0.5; (**B**) *c* = 2 wt%.

Since Pickering emulsions that are stabilized with LF–FD complexes are opaque liquids, the ΔBS of the emulsions at different heights with time was measured. As shown in Figure 9, the ΔBS at the bottom of the Pickering emulsion stabilized by the LF–FD complexes increased slightly with time due to the slight precipitation at the bottom of the emulsion, which caused a decrease in transmitted light and an increase in scattered light [24]. In contrast, the change in ΔBS at the top of the emulsion was exactly the opposite. The above phenomena indicated that the droplets of the Pickering emulsions underwent agglomeration during the time measured, resulting in a certain degree of emulsion delamination, which was caused by the fact that freshly prepared emulsions were highly susceptible to a drop in the emulsion phase during initial storage.

According to Figure 9A, the ΔBS profile decreased significantly with increasing LF–FD complex concentration at the same oil phase ratio (*φ* = 0.5). This was because the increase in LF–FD complex concentration could promote the formation of a denser interfacial layer, which could stabilize more emulsion droplets and increase the density of the emulsion droplets. The increase in emulsion droplet density meant that the number of interdroplet collisions increased, which was beneficial for prolonging the adsorption time for the LF–FD complex to cover the oil droplets, thus effectively avoiding the aggregation of the emulsion droplets [46]. According to Figure 9B, it could be found that at the same concentration (*c* = 2 wt%) of LF–FD complex, the ΔBS profile decreased significantly with increasing oil phase ratio because the increase in oil phase ratio made the emulsion viscosity increase accordingly, thus limiting the droplet movement in the Pickering emulsion. The effect of LF–FD complex concentration and oil phase ratio on ΔBS was consistent with the TSI trend.

#### 3.2.7. Rheological Properties of Pickering Emulsions

**Figure 10:**
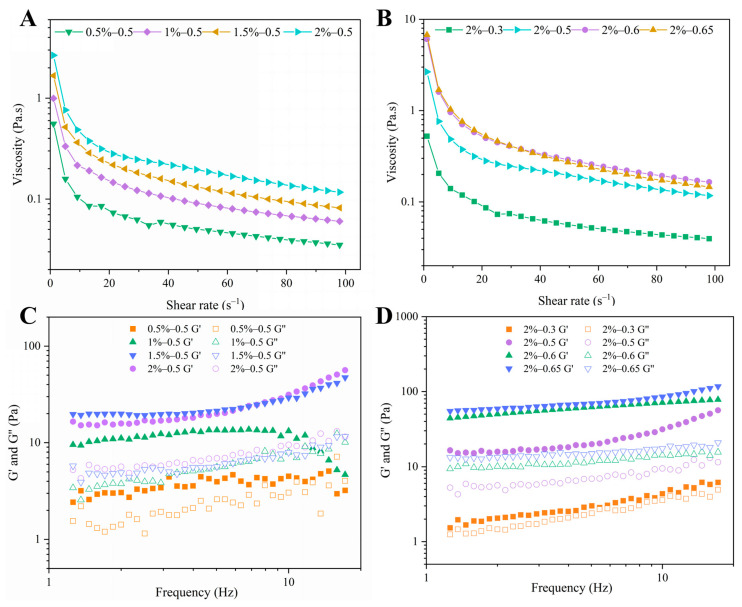
Viscosity of Pickering emulsions stabilized with different *c* (0.5–2 wt%) of LF–FD complexes at different *φ* (0.3–0.65): (**A**) *φ* = 0.5; (**B**) *c* = 2 wt%; storage modulus (G′) and loss modulus (G″) of Pickering emulsions stabilized with different c (0.5–2 wt%) of LF–FD complexes at different *φ* (0.3–0.65): (**C**) *φ* = 0.5; (**D**) *c* = 2 wt%.

The rheological properties of emulsions can be explored to further investigate the stability and microstructural complexity of emulsions [47]. From Figure 10A,B, it can be seen that the viscosity of the Pickering emulsions stabilized with the LF–FD complexes decreased with increasing shear rate and exhibited shear-thinning behavior. As shown in Table 2, *n* was less than 1, which indicated that the emulsions were all pseudoplastic flows of non-Newtonian fluids [48]. This could be because the distribution of the emulsion droplets was broken by high-speed shear, which rearranged the droplets in the flow direction, resulting in a reduction in the resistance of emulsion droplet flow [22]. Among them, the higher the concentration of the LF–FD complexes, the higher the viscosity of the Pickering emulsions, which might be caused by the fact that the concentration of the LF–FD complexes was so high that it exceeded the saturation coverage of the oil droplet surface. The interaction between the free LF–FD complexes in the aqueous phase increased the viscosity of the emulsion [49]. Similarly, the higher the ratio of the oil phase, the higher the viscosity of the Pickering emulsion. This was because the increase in the ratio of the oil phase made the droplets larger and reduced the water phase, resulting in a denser interdroplet arrangement and an increase in viscosity [50,51]. Moreover, the *K* value of the emulsion increased with the increase in LF-FD complex concentration and oil phase ratio, and *n* showed an opposite trend. Thus, the Pickering emulsion stabilized with the LF–FD complex (*c* = 2 wt%, *φ* = 0.65) had the largest *K* value and the smallest *n* value, indicating that this Pickering emulsion had a good apparent viscosity, which was conducive to enhancing the stability of the emulsion. This was consistent with the results of stability studies.

According to Figure 10C,D, the storage modulus (G′) and loss modulus (G″) of Pickering emulsions stabilized with LF–FD complexes increased with frequency to different degrees except for the 0.5%–0.5 and 1%–0.5 groups, and G′ was higher than G″, which indicated that the Pickering emulsions exhibited solid-like behavior in the linear viscoelastic region [52]. In contrast, as the frequency increased, G″ was higher than G′ in the 0.5%–0.5 and 1%–0.5 groups, changing the structure of the emulsions with increasing frequency, giving them a liquid behavior. Furthermore, the G′ and G″ of the Pickering emulsions increased with LF–FD complex concentration and oil phase ratio. This was due to the rise in the concentration of the complex, which led to a smaller emulsion droplet size, which, in turn, resulted in a tighter interdroplet structure. The increase in the proportion of the oil phase resulted in a tighter accumulation of emulsion droplets [50]. As a result, the deformation capacity of the emulsion increased, and thus, the modulus increased. The above results are consistent with the change in viscosity, indicating that the rheological properties of the emulsion were changed by varying the LF–FD complex concentration and oil phase ratio.

## 4. Conclusions

LF–FD complexes were prepared and used to stabilize Pickering emulsions, and the stability and rheological properties were investigated. The results showed that LF–FD complexes with good wettability were mainly driven by electrostatic interactions. They could effectively adsorb on the surface of oil droplets to form an interfacial layer, which was beneficial to the preparation of a Pickering emulsion. Pickering emulsions with higher oil–phase ratios and stabilized with high concentrations of LF–FD complexes had better stability. This was because the high concentration of LF–FD complex reduced the droplet size, and the high oil content allowed for tighter droplet accumulations. This study provides a new approach for preparing protein–polysaccharide-stabilized food-grade Pickering emulsions. Besides, the realization of encapsulation using a high concentration of clove oil increases the scope of its application. Subsequently, this Pickering emulsion will be used to prepare emulsified films to extend the shelf life of food products.

## Figures and Tables

**Table 1:** Emulsion stability index of Pickering emulsions stabilized by LF–FD particles. The data are expressed as means ± SD (*n* = 3). Different letters in the same line indicate significant differences (*p* < 0.05).

Samples	Emulsion Stability Index (%)			
	0 d	7 d	15 d	30 d
0.5%–0.3	68.28 ± 2.48 ^a^	62.92 ± 2.32 ^b^	60.97 ± 1.88 ^b^	60.97 ± 1.88 ^b^
0.5%–0.5	76.67 ± 2.18 ^a^	73.33 ± 1.65 ^b^	70.48 ± 0.82 ^bc^	68.10 ± 0.82 ^c^
0.5%–0.6	91.27 ± 2.76 ^a^	85.00 ± 1.44 ^a^	83.00 ± 2.47 ^a^	83.00 ± 2.47 ^a^
0.5%–0.65	92.03 ± 1.49 ^a^	91.51 ± 0.58 ^a^	88.53 ± 1.98 ^b^	88.51 ± 0.48 ^b^
1%–0.3	86.93 ± 3.61 ^a^	75.49 ± 1.70 ^b^	66.02 ± 1.49 ^c^	64.08 ± 2.21 ^c^
1%–0.5	88.38 ± 2.73 ^a^	77.68 ± 1.54 ^b^	75.74 ± 1.26 ^b^	62.16 ± 2.38 ^b^
1%–0.6	92.69 ± 2.33 ^a^	88.23 ± 0.35 ^b^	86.27 ± 1.76 ^b^	76.99 ± 1.60 ^c^
1%–0.65	95.51 ± 1.43 ^a^	89.01 ± 1.66 ^b^	87.51 ± 1.34 ^b^	87.51 ± 0.64 ^b^
1.5%–0.3	88.35 ± 2.95 ^a^	71.85 ± 1.51 ^b^	69.40 ± 2.82 ^b^	69.40 ± 2.82 ^b^
1.5%–0.5	97.44 ± 0.89 ^a^	90.26 ± 0.76 ^b^	88.72 ± 0.74 ^bc^	88.72 ± 0.74 ^c^
1.5%–0.6	100.00 ± 0.00 ^a^	92.93 ± 1.75 ^b^	91.92 ± 0.87 ^b^	91.92 ± 0.87 ^b^
1.5%–0.65	100.00 ± 0.00 ^a^	100.00 ± 0.00 ^a^	93.63 ± 0.85 ^b^	93.63 ± 0.85 ^b^
2%–0.3	99.05 ± 1.65 ^a^	78.63 ± 1.89 ^b^	77.68 ± 1.54 ^b^	75.73 ± 2.21 ^b^
2%–0.5	97.58 ± 4.18 ^a^	88.44 ± 3.79 ^b^	87.39 ± 3.28 ^b^	87.39 ± 3.28 ^b^
2%–0.6	100.00 ± 0.00 ^a^	92.16 ± 1.70 ^b^	92.16 ± 1.70 ^b^	89.22 ± 1.70 ^b^
2%–0.65	100.00 ± 0.00 ^a^	100.00 ± 0.00 ^a^	100.00 ± 0.00 ^a^	99.61 ± 0.68 ^a^

**Table 2:** Power law equations of Pickering emulsions stabilized with LF–FD complexes. The data are expressed as means ± SD (*n* = 3).

Samples	*K* (Pa·s^n^)	*n*	*R* ^2^
0.5%–0.5	0.535 ± 0.004	0.413 ± 0.003	0.995
1%–0.5	1.053 ± 0.013	0.351 ± 0.008	0.996
1.5%–0.5	1.778 ± 0.018	0.367 ± 0.004	0.998
2%–0.5	2.843 ± 0.040	0.262 ± 0.116	0.995
2%–0.3	0.556 ± 0.004	0.405 ± 0.003	0.999
2%–0.6	6.543 ± 0.036	0.167 ± 0.004	0.998
2%–0.65	7.340 ± 0.026	0.133 ± 0.003	0.999

## Data Availability

The data herein presented are available on request from the corresponding author.

## Supplementary Materials

The following supporting information can be downloaded at: https://www.mdpi.com/article/10.3390/polym15081820/s1, Figure S1: The visual appearance of LF, FD, and LF–FD complexes (1:1, 1.5:1 and 2:1) before and after heating at different pHs; Figure S2: Different phase areas at m(LF):m(FD) = 2:1; Figure S3: Static water-in-air contact angles of heated LF; Figure S4: Dispersibility of Pickering emulsions in water (A) and oil (B); Figure S5: Appearance of Pickering emulsions stabilized with different *c* (0.5–2 wt%) of LF–FD complexes at different *φ* (0.3–0.65). Photographs were taken at 0–30 days after the preparation of fresh emulsions; Figure S6: Centrifugal stability of Pickering emulsions stabilized with different *c* (0.5–2 wt%) of LF–FD complexes at different *φ* (0.3–0.65).
