# Supplementary material for: Clove Essential Oil Pickering Emulsions Stabilized with Lactoferrin/Fucoidan Complexes: Stability and Rheological Properties
Source: Polymers (Basel). 2023 Apr 7;15(8):1820. doi: 10.3390/polym15081820 (PMC10143265; doi:10.3390/polym15081820)
Supplement: Supplementary file 1 [file polymers-15-01820-s001.zip › polymers-2281892-supplementary.pdf]

Supplementary Information

for

Clove Essential Oil Pickering Emulsions Stabilized with Lactoferrin/Fucoidan Complexes: Stability and Rheological Properties

Xiaohong Xi [1], Zihao Wei [1,*], Yanan Xu [1] and Changhu Xue [1,2]

[1] College of Food Science and Engineering, Ocean University of China, Qingdao 266404, China
[2] Laboratory of Marine Drugs and Biological Products, Pilot National Laboratory for Marine Science and Technology, Qingdao 266237, China
* Correspondence: weizihao@ouc.edu.cn

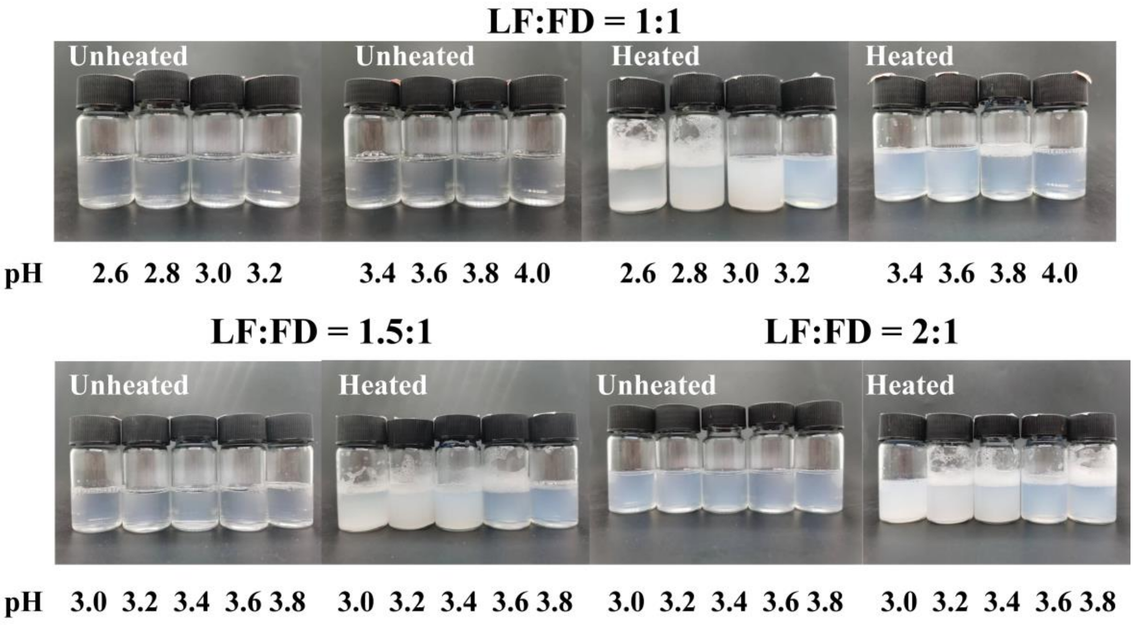

Figure S1. The visual appearance of LF, FD and LF-FD complexes (1:1, 1.5:1 and 2:1) before and after heating at different pH.

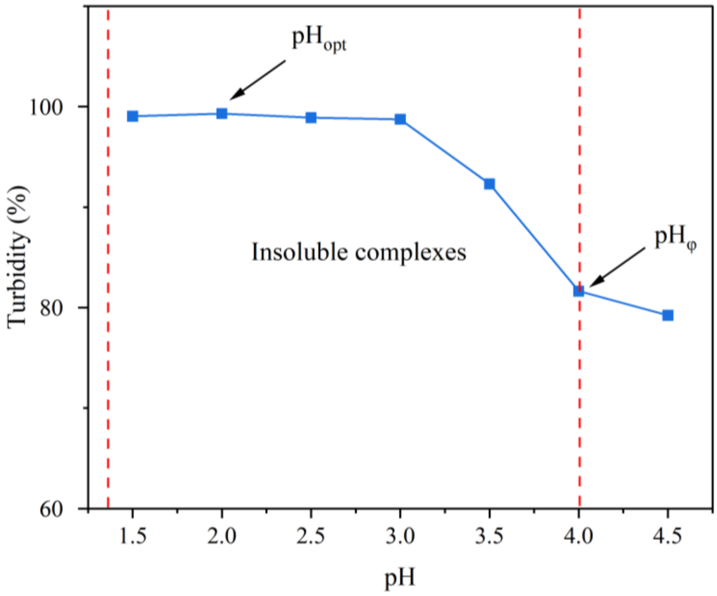

Figure S2. Different phase areas at m(LF) : m(FD) = 2:1.

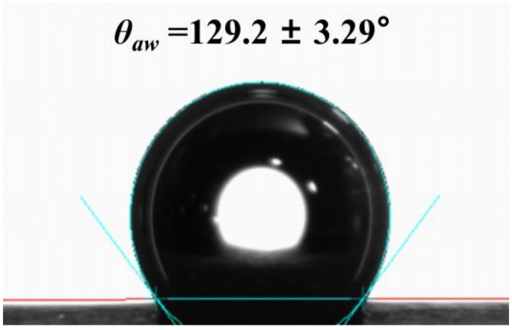

Figure S3. Static water-in-air contact angles of heated LF.

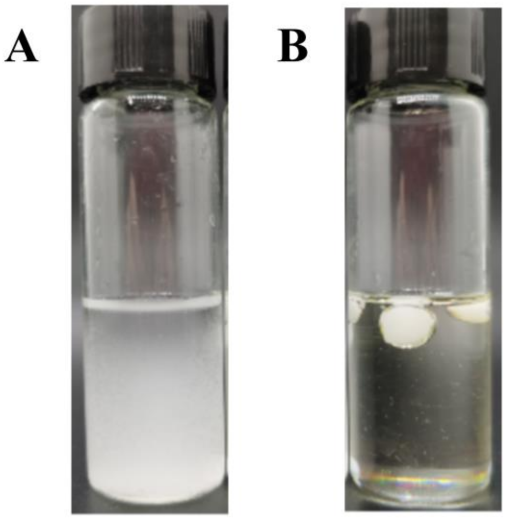

Figure S4. Dispersibility of Pickering emulsions in water (A) and oil (B).

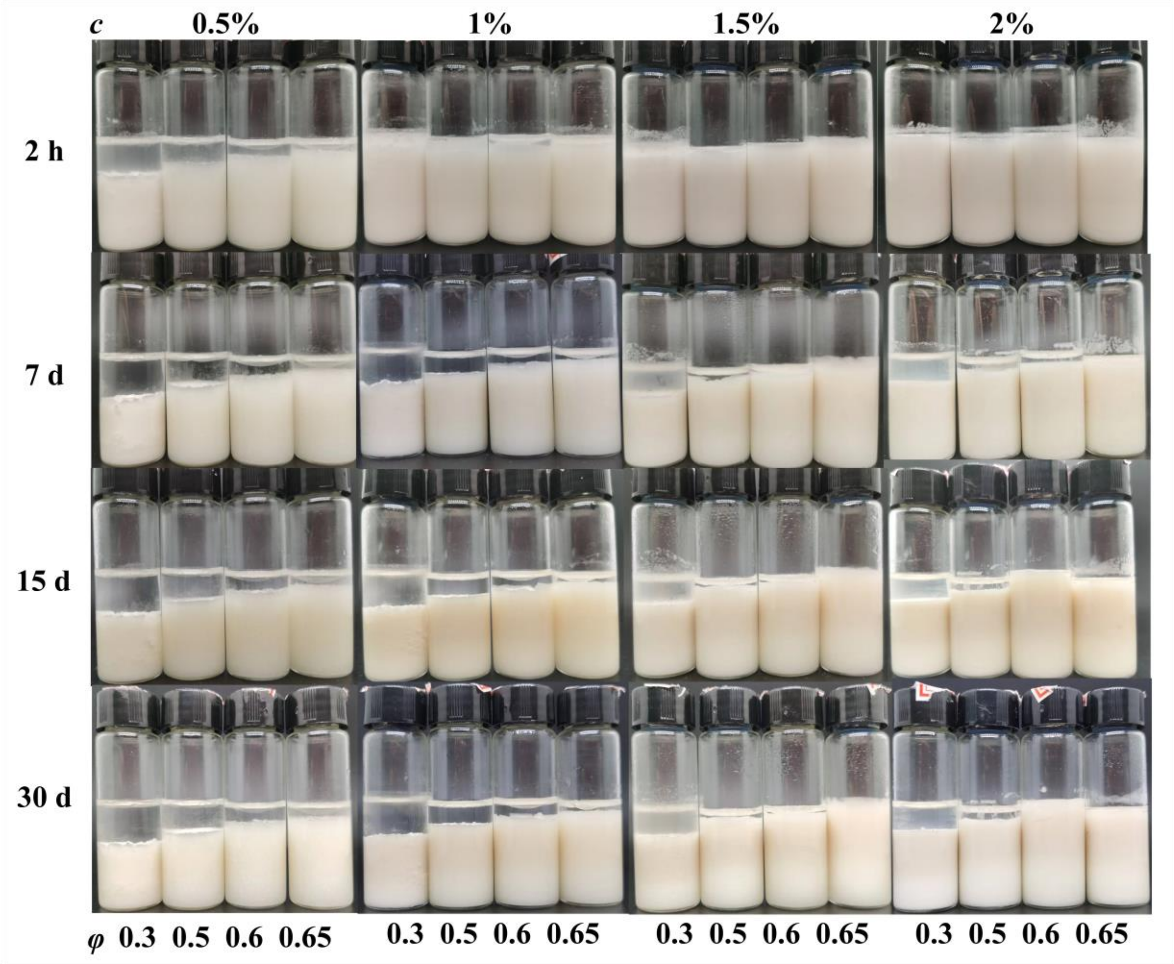

Figure S5. Appearance of Pickering emulsions stabilized by different *c* (0.5−2 wt%) of LF−FD complexes at different *φ* (0.3−0.65). Photographs were taken at 0−30 days of preparation of fresh emulsions.

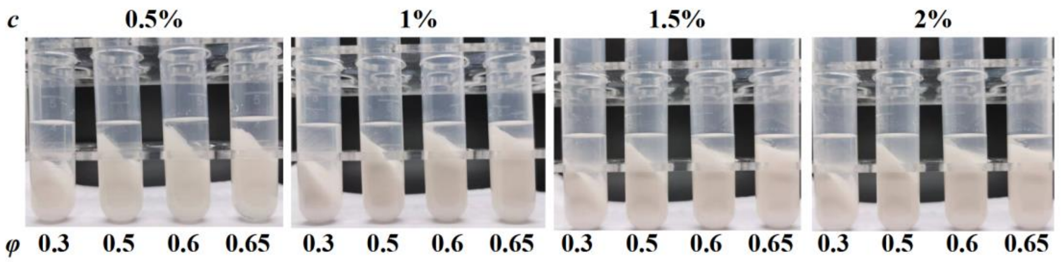

Figure S6. Centrifugal stability of Pickering emulsions stabilized by different c (0.5−2 wt%) LF−FD complexes at different φ (0.3−0.65).
